# Effect of secretory pathway gene overexpression on secretion of a fluorescent reporter protein in *Aspergillus nidulans*

**DOI:** 10.1186/s40694-016-0021-y

**Published:** 2016-04-12

**Authors:** Martin Schalén, Diana Chinyere Anyaogu, Jakob Blæsbjerg Hoof, Mhairi Workman

**Affiliations:** Department of Systems Biology, Technical University of Denmark, Søltofts Plads, Building 223, 2800 Kgs. Lyngby, Denmark

**Keywords:** Secretory pathway, *Aspergillus nidulans*, Fluorescent reporter

## Abstract

**Background:**

The considerable capacity of filamentous fungi for the secretion of proteins is the basis for multi-billion dollar industries producing enzymes and proteins with therapeutic value. The stepwise pathway from translation to secretion is therefore well studied, and genes playing major roles in the process have been identified through transcriptomics. The assignment of function to these genes has been enabled in combination with gene deletion studies. In this work, 14 genes known to play a role in protein secretion in filamentous fungi were overexpressed in *Aspergillus nidulans*. The background strain was a fluorescent reporter secreting mRFP. The overall effect of the overexpressions could thus be easily monitored through fluorescence measurements, while the effects on physiology were determined in batch cultivations and surface growth studies.

**Results:**

Fourteen protein secretion pathway related genes were overexpressed with a tet-ON promoter in the RFP-secreting reporter strain and macromorphology, physiology and protein secretion were monitored when the secretory genes were induced. Overexpression of several of the chosen genes was shown to cause anomalies on growth, micro- and macro-morphology and protein secretion levels. While several constructs exhibited decreased secretion of the model protein, the overexpression of the Rab GTPase RabD resulted in a 40 % increase in secretion in controlled bioreactor cultivations. Fluorescence microscopy revealed alterations of protein localization in some of the constructed strains, giving further insight into potential roles of the investigated genes.

**Conclusions:**

This study demonstrates the possibility of significantly increasing cellular recombinant protein secretion by targeted overexpression of secretion pathway genes. Some gene targets investigated here, including genes from different compartments of the secretory pathway resulted in no significant change in protein secretion, or in significantly lowered protein titres. As the 14 genes selected in this study were previously shown to be upregulated during protein secretion, our results indicate that increased expression may be a way for the cell to slow down secretion in order to cope with the increased protein load. By constructing a secretion reporter strain, the study demonstrates a robust way to study the secretion pathway in filamentous fungi.

**Electronic supplementary material:**

The online version of this article (doi:10.1186/s40694-016-0021-y) contains supplementary material, which is available to authorized users.

## Background

Filamentous fungi have a naturally high protein secretion capacity. Therefore, they are interesting hosts for production of industrially relevant enzymes and therapeutic proteins. Approximately 50 % of industrial enzymes are produced in filamentous fungi, with production levels reported to be as high as tens of grams per liter [1]. Production levels with proteins of non-fungal origin are often disappointingly low, typically in the milligram per liter range. The reasons for this phenomenon are relatively poorly understood, but it seems that the limitations are at the post-transcriptional level with bottlenecks occurring due to compartmentalisation or at stages in the processing of the protein for secretion [2].

Several studies have attempted to shed light on the extraordinary secretion capacity of filamentous fungi, primarily at the transcriptomic level [3–6]. These studies have led to the identification of genes that play major roles in the different stages of protein secretion such as translocation, folding, cargo transport and exocytosis. In combination with gene deletion studies, the functionality and importance of some secretion related genes have been characterized in more depth. For example, the *Aspergillus niger* Rab GTPase *srgA* (*SEC4* in *Saccharomyces cerevisiae*, *rabD* in *Aspergillus nidulans*) has been shown to have a role in protein secretion, but is not required for survival [7]. Recently, Kwon et al [8] created *in vivo* reporter strains to study the trafficking and dynamics of secretory vesicles in *A. niger* and highlighted gene-specific differences between the secretory pathways of *S. cerevisiae* and *A. niger*.

Transport through the secretory pathway begins with translocation of the protein to the ER, where the protein is glycosylated, phosphorylation occurs and disulfide bridges are formed. After passing a sophisticated quality control mechanism, the cargo is transported in vesicles from the ER to the Golgi apparatus. The vesicles bud off from the ER membrane and tether to the Golgi with the aid of soluble *N*-ethylmaleimide-sensitive (NSF) factor receptor (SNARE) that mediates vesicle docking and fusion [9]. After further modifications in the Golgi apparatus, such as glycosylation and peptide processing, the secretory cargo leaves the Golgi in vesicles bound for the plasma membrane, where exocytosis occurs. The secretory pathway in yeast and filamentous fungi is described in detail in several reviews [2, 10–15].

Typically, studies on the secretory pathway in filamentous fungi involve the deletion of genes to investigate the role or effect of that gene product, whereas the strategy of using overexpression of genes in filamentous fungi is not as frequent as in *S. cerevisiae.* A recent example of engineering the secretory pathway in *S. cerevisiae* is the overexpression of two Sec1/Munc18 (SM) proteins involved in different transport steps [16]. SM proteins assist in SNARE complex formation for vesicle fusion. Overexpression of *SEC1* was shown to cause increased secretion of insulin and α-amylase, whereas overexpression of *SLY1* only increased the secretion of α-amylase. The study showed that engineering single genes in the secretion pathway may be an efficient strategy to improve protein secretion, but also that results depend on characteristics of the protein to be secreted.

A common approach for secreting heterologous proteins in filamentous fungi is fusion of the heterologous protein to a known, well-secreted, native protein and this strategy has been extensively used for studying the process of protein secretion [17–19]. Gordon et al. [17] employed this technique in order to study protein secretion in vivo. GFP was fused to glucoamylase, and protein secretion was shown to localize to the hyphal tips. Reporter strains expressing fluorescent proteins are interesting as they give several possibilities of analysis, for example microscopy for single cell studies and fluorescence measurements for quantitative studies.

**Fig. 1:**
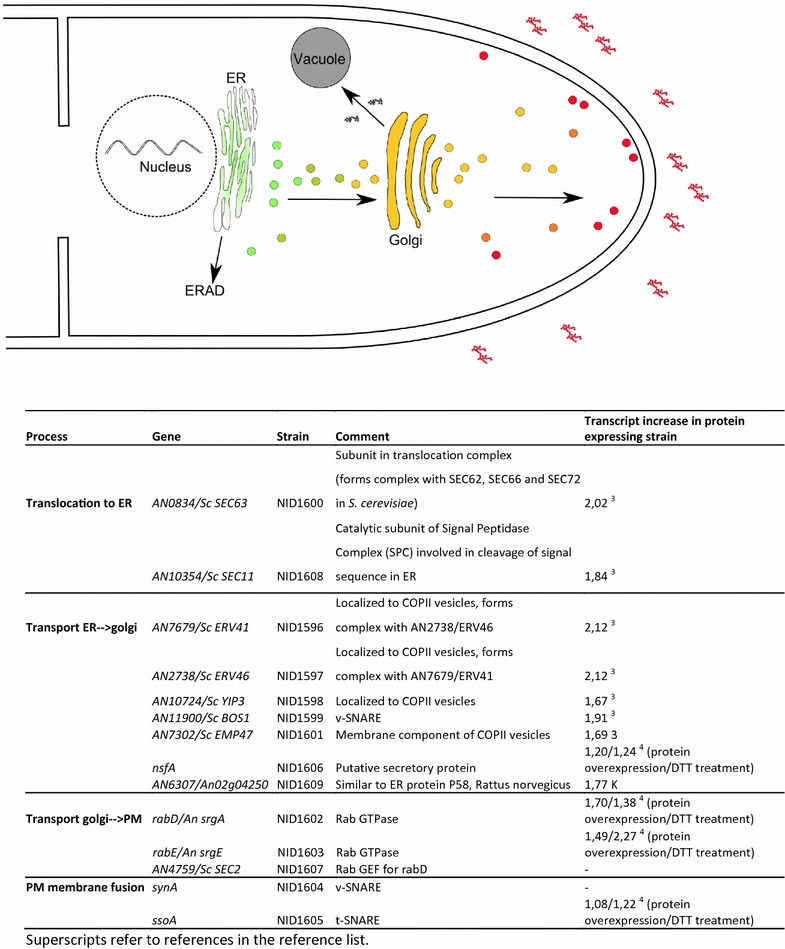
Protein secretion pathway in Aspergilli and genes overexpressed in the secretory pathway of *A. nidulans*. For visualization the Golgi is stacked, although this is not the case in *A. nidulans* [31]. AN, *Aspergillus nidulans*; An, *Aspergillus* niger; Sc, *Saccharomyces* cerevisiae

In the current study, the effects of manipulating the secretion pathway in *A. nidulans* have been characterised using a fluorescent reporter in *A. nidulans*. The reporter strain created utilizes mRFP fused to the first 514 amino acids of glucoamylase (GlaA_1–514_) from *A. niger* as a carrier protein. This reporter strain has been used as the background strain for construction of 14 strains that overexpress different genes known to have roles in the secretion pathway. The strains have been constructed in a manner that allows overexpression of the secretory genes to be induced by doxycycline, making it possible to study the effect of a variable overexpression of the relevant gene [20]. An overview presenting the selected genes in relation to their localisation in the fungal hyphal compartments is shown in Fig. 1. The genes have been selected on the basis of existing knowledge from studies investigating the effect of protein overexpression on the transcriptome of filamentous fungi and *Saccharomyces cerevisiae* [3–6, 21]. Some of the chosen genes are part of complex structures, such as COPII vesicles, whereas others have targeted modes of action, such as fusion of vesicles to the plasma membrane. Importantly, the selection of genes was chosen from several parts of the secretory pathway, covering different compartments and processes (translocation to ER, transport to Golgi, intra-Golgi transport, Golgi to plasma membrane transport and vesicle fusion at the plasma membrane) in order to understand how transport of the secretory cargo through the cell can be improved.

## Results and discussion

### Evaluation of reporter strain

**Fig. 2:**
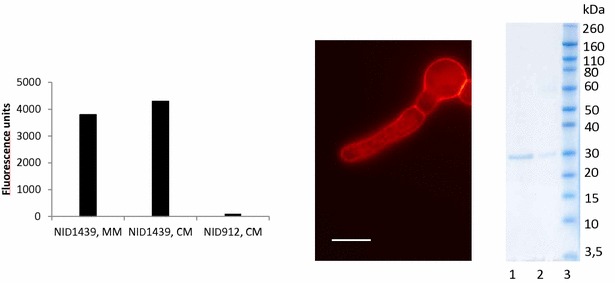
Validation of the secretion reporter. *Left* Liquid cultures of NID1439 grown for 48 h at 30 °C without shaking. *Middle* Fluorescence microscopy of NID1439. Fluorescence localizes to plasma membrane, septa and ER-like structures. *Right* SDS-PAGE of His-purified (1) mRFP, cell culture supernatant from CM (2) and ladder (3). *Scale bar* 10 µm

To be able to compare the characteristics of different secretory pathway mutants an *A. nidulans* strain secreting the model protein mRFP was constructed. A *glaA*_1–514_-fused mRFP construct was integrated in the genome of NID1, and verified by spore PCR. To release the mRFP model protein from the glucoamylase, a KEX2 site (Lys–Arg) for proteolytic processing in the Golgi was inserted between the fused proteins. This reporter strain, NID1439, was screened for protein secretion by microscopy, liquid cultures and SDS-PAGE (Fig. 2). Microscopy showed fluorescence predominantly localizing to hyphal tips, plasma membrane and septa, as expected for a protein in the process of secretion [17, 22, 23]. This finding was backed by fluorescence measurements of the supernatant from 48 h liquid cultures which confirmed that the protein was secreted. Fluorescence was around 4000 units, whereas for the control strain expressing an intracellular mRFP (NID912), no fluorescence was detected in the supernatant. Due to fluorescence at intracellular structures there was a possibility that some of the secretory cargo was trapped inside the cell. Finally, proteins bearing a His-tag were purified and subsequent SDS-PAGE of the cell culture supernatant and the His-purified protein demonstrated that mRFP (28 kDa band) was secreted from the cells, and inherently that it was efficiently cleaved from the glucoamylase gene, since no band corresponding to glucoamylase fused mRFP (approximately 85 kDa) was seen. A very faint band at around 60 kDa can be seen, and this corresponds to the size of the glucoamylase cleaved off from the mRFP peptide.

### Construction of secretory mutants and initial observations

**Fig. 3:**
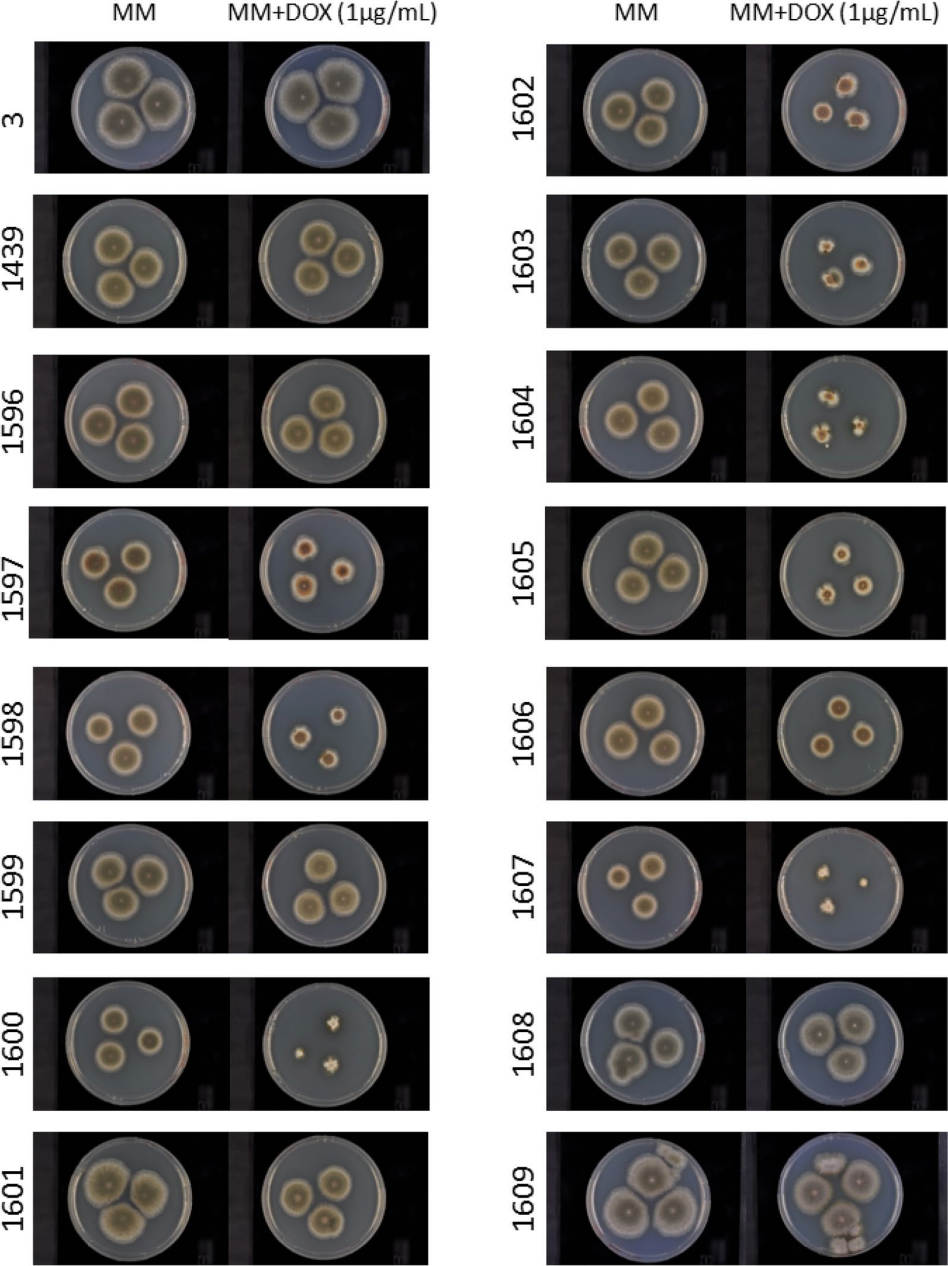
Growth experiments for all strains with modifications in the secretory pathway. Strains were cultivated on MM and MM + DOX (1 μg/mL). GlaA-RFP refers to the reference strain NID1439, with no modification of secretion related genes

The auxotrophy for uridine and uracil was regenerated by plating NID1439 on MM + 5-FOA, thus generating strain NID1595 where the AF*pyrG* had looped out by direct-repeat recombination. Fourteen genes (see Fig. 1) were chosen for overexpression using the doxycycline inducible tet-ON promoter. The genes were transformed in to NID1595, resulting in strains NID1596-NID1609, see Table 1. Each of the strains was rigorously verified (by spore PCR) for integration of the secretory genes in IS1. The integration site was characterized in an earlier study [24]. Furthermore, southern blot of some chosen strains was performed in order to rule out random integration in the genome (Additional file 1: Figure 1). All strains were plated on MM and MM supplemented with DOX (1 μg/mL) to study growth and any morphological effect of overexpressing the secretory genes. None of the strains had an altered growth when no DOX was present, however upon induction by DOX nine out of 14 strains demonstrated decreased radial growth (NID1597-98, 1600, 1602-07) (Fig. 3). These nine strains individually overexpress genes in different compartments, so no general trends in decreased growth relating to an alteration of a specific compartment could be seen. Furthermore, altered morphology when DOX was added demonstrated that it was indeed overexpression of the specific secretion related gene causing the effect. If integration had occurred at additional sites and this would affect the strain, the effect would be seen also when DOX was not added to the plates.

**Table 1:** Strains used in this study

Name	Genotype	Gene(s) affected	Description	Source
NID1	*argB2, pyrG89, veA1, nkuA*Δ	nkuAΔ for efficient gene targeting	Parental strain used to construct mRFP secreting strain	IBT collection #29539 [33]
NID3	*argB2, pyrG89, veA1, nkuA*-*trS::AFpyrG*	Transient small repeat in *nkuA*	Reference strain	IBT collection #28738 [33]
NID912	*argB2*, *pyrG89, veA1*, *nkuA*Δ, IS1::PgpdA::RFP::TtrpC::*pyrG*	Intracellular mRFP expression	Negative control	Our lab
NID1439	*argB2, pyrG89, veA1, nkuA*Δ, *IS1::PgpdA*-*ASNglaA*-*mRFP*-*TtrpC*::AF*pyrG*	mRFP secretion	Strain secreting mRFP	This study
NID1595	*argB2, pyrG89, veA1, nkuA*Δ, *IS1::PgpdA*-*ASNglaA*-*mRFP*-*TtrpC*	mRFP secretion	Strain secreting mRFP, parental strain for NID1596-NID1609	This study
NID1596	*argB2, pyrG89, veA1, nkuAΔ, IS5::PgpdA*-*ASNglaA*-*mRFP*-*TtrpC, IS1::PtetON*-AN7679-*TtrpC::AFpyrG*	mRFP secretion, AN7679 overexpressed	Sc *ERV41* ortholog	This study
NID1597	*argB2, pyrG89, veA1, nkuA*Δ*, IS5::PgpdA*-*ASNglaA*-*mRFP*-*TtrpC, IS1::PtetON*-AN2738-*TtrpC::AFpyrG*	mRFP secretion, AN2738 overexpressed	Sc *ERV46* ortholog	This study
NID1598	*argB2, pyrG89, veA1, nkuA*Δ*, IS5::PgpdA*-*ASNglaA*-*mRFP*-*TtrpC, IS1::PtetON*-AN10724-*TtrpC::AFpyrG*	mRFP secretion, AN10724 overexpressed	Sc *YIP3* ortholog	This study
NID1599	*argB2, pyrG89, veA1, nkuA*Δ*, IS5::PgpdA*-*ASNglaA*-*mRFP*-*TtrpC, IS1::PtetON*-AN11900-*TtrpC::AFpyrG*	mRFP secretion, AN11900 overexpressed	Sc *BOS1* orholog	This study
NID1600	*argB2, pyrG89, veA1, nkuA*Δ*, IS5::PgpdA*-*ASNglaA*-*mRFP*-*TtrpC, IS1::PtetON*-AN0834-*TtrpC::AFpyrG*	mRFP secretion, AN0834 overexpressed	AN *sec63*	This study
NID1601	*argB2, pyrG89, veA1, nkuA*Δ*, IS5::PgpdA*-*ASNglaA*-*mRFP*-*TtrpC, IS1::PtetON*-AN7302-*TtrpC::AFpyrG*	mRFP secretion, AN7302 overexpressed	Sc *EMP47* ortholog	This study
NID1602	*argB2, pyrG89, veA1, nkuA*Δ*, IS5::PgpdA*-*ASNglaA*-*mRFP*-*TtrpC, IS1::PtetON*-*ANRabD*-*TtrpC::AFpyrG*	mRFP secretion, *ANrabD* overexpressed	AN *rabD*	This study
NID1603	*argB2, pyrG89, veA1, nkuA*Δ*, IS5::PgpdA*-*ASNglaA*-*mRFP*-*TtrpC, IS1::PtetON*-*ANRabE*-*TtrpC::AFpyrG*	mRFP secretion, *ANrabE* overexpressed	AN *rabE*	This study
NID1604	*argB2, pyrG89, veA1, nkuA*Δ*, IS5::PgpdA*-*ASNglaA*-*mRFP*-*TtrpC, IS1::PtetON*-*ANSynA*-*TtrpC::AFpyrG*	mRFP secretion, *ANsynA* overexpressed	AN *synA*	This study
NID1605	*argB2, pyrG89, veA1, nkuA*Δ*, IS5::PgpdA*-*ASNglaA*-*mRFP*-*TtrpC, IS1::PtetON*-*ANSsoA*-*TtrpC::AFpyrG*	mRFP secretion, *ANssoA* overexpressed	AN *ssoA*	This study
NID1606	*argB2, pyrG89, veA1, nkuA*Δ*, IS5::PgpdA*-*ASNglaA*-*mRFP*-*TtrpC, IS1::PtetON*-*ANNsfA*-*TtrpC::AFpyrG*	mRFP secretion, *ANnsfA* overexpressed	AN *nsfA*	This study
NID1607	*argB2, pyrG89, veA1, nkuA*Δ*, IS5::PgpdA*-*ASNglaA*-*mRFP*-*TtrpC, IS1::PtetON*-AN4759-*TtrpC::AFpyrG*	mRFP secretion, AN4759 overexpressed	Sc *SEC2* ortholog	This study
NID1608	*argB2, pyrG89, veA1, nkuA*Δ*, IS5::PgpdA*-*ASNglaA*-*mRFP*-*TtrpC, IS1::PtetON*-AN10354-*TtrpC::AFpyrG*	mRFP secretion, AN10354 overexpressed	S.c. *SEC11* ortholog	This study
NID1609	*argB2, pyrG89, veA1, nkuA*Δ*, IS5::PgpdA*-*ASNglaA*-*mRFP*-*TtrpC, IS1::PtetON*-AN6307-*TtrpC::AFpyrG*	mRFP secretion, AN6307 overexpressed	*An02g04250* ortholog	This study

AN, *Aspergillus nidulans*; An, *Aspergillus niger*; AF, *Aspergillus fumigatus*; Sc, *Saccharomyces cerevisiae*

### Protein secretion and macromorphology

**Fig. 4:**
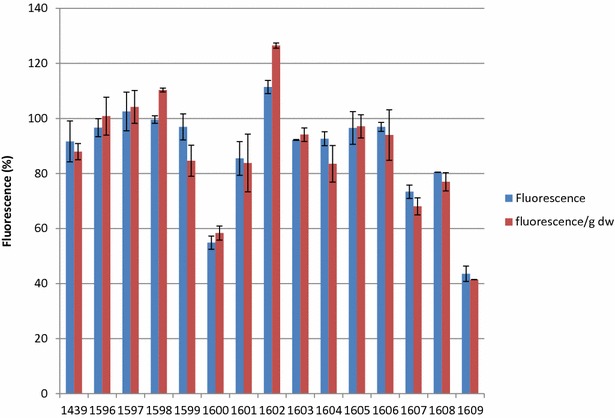
Protein secretion as measured by fluorescence in culture supernatant in shake flasks cultivations. Shake flasks were performed in duplicate, at 150 rpm and 30 °C, and the bars depict the maximum fluorescence level in the strain when DOX (10 μg/mL) was added compared to non-induced conditions (±one standard error). *Blue bars* fluorescence, *Red bars* fluorescence per g dry weight

 With the aim of investigating how overexpression of the selected genes affected the secretion of the model protein, submerged cultivations were used as a basis for providing quantitative measurements of cellular physiology parameters. The strains were cultivated in shake flasks and fluorescence levels and cell dry weight were measured over time. The medium was supplemented with MES buffer in order to avoid pH related effects on the fluorescent signal. Figure 4 shows how the maximum mRFP fluorescence change due to induction of the individual secretion related genes (DOX concentration 10 μg/mL). Secretion of mRFP was slightly decreased in the control strain when DOX was added. However, as total biomass (measured as maximum dry weight) was comparable with and without DOX, it was assumed that DOX had no significant negative effect on growth [20]. Furthermore, the maximum fluorescence was reached at the same time with and without DOX. As we wanted to examine the effect on secretory production of a recombinant protein by engineering the secretory pathway, we decided to use the maximum fluorescence values reached in each cultivation, rather than collecting samples after a certain time point. The maximum fluorescence levels were in general reached at similar time points, however differences within a few hours occurred between strains.

**Fig. 5:**
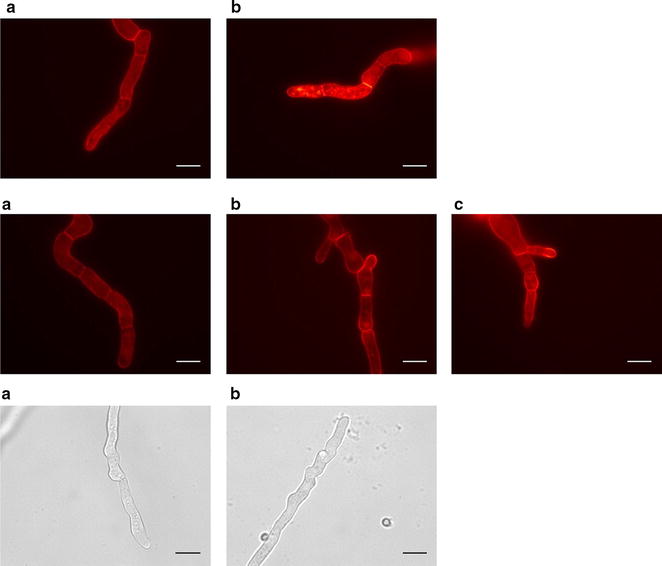
Fluorescence microscope images of NID1600 and NID1602. Spores were inoculated on MM and MM + DOX (1 μg/mL) slides and incubated at 30 °C in petri dishes. *Top* NID1600 (AN0834 overexpressed) non-induced (**a**) and induced (**b**). ER-like structures were more pronounced when the secretory gene was induced. *Middle* NID1602 (ANrabD overexpressed) non-induced (**a**) and induced (**b** and **c**). Fluorescence increases at plasma membrane when *rabD* is overexpressed. *Bottom* Stunted growth of NID1602 induced with DOX (*left* and *right*). *Scale bar* 10 µm

To increase the flux of proteins to ER *A. nidulans* *Sec63 (*AN0834) and the ortholog of *S. cerevisiae SEC11* (AN10354) were overexpressed (generating strains NID1600 and NID1608, respectively). Overexpression of *SEC63* resulted in significantly lower fluorescence, approximately 40 % decrease, whereas *SEC11* overexpression resulted in a slight decrease in secretion. Moreover, intracellular fluorescence in ER-like structures was seen in NID1600 induced with DOX (Fig. 5) and this strain exhibited a substantially decreased radial growth on solid MM + DOX, indicating a stress response when the gene was overexpressed. However, decreased radial growth on plates does not per se relate to a decreased secretory capacity. NID1609 was the strain secreting the least mRFP of all strains investigated having approximately 40 % of the secretory capacity compared to non-induced conditions, which was not reflected on solid media, as growth was stronger than e.g. NID1602 on MM + DOX, albeit with a different morphology. In NID1609, an ortholog of *A. niger* An02g04250 (AN6307) was overexpressed. An02g04250 is similar to the ER chaperone P58 in *Rattus norvegicus*, a rat homolog of human ERGIC-53 [3]. In humans this protein is involved in glycoprotein sorting between the ER and the Golgi [25]. A possible explanation for the decreased secretion in NID1600 might be an overload of the ER which can result in Endoplasmatic Reticulum Associated Degradation (ERAD) [26].

### Genes involved in ER to golgi transport

In *S. cerevisiae, ERV41* and *ERV46* are localized to COPII vesicles where they form a complex. The overexpression of the *ERV41* ortholog, AN7679, in *A. nidulans* (NID1596) resulted in markedly different phenotypic behaviour on plates compared to *ERV46* (AN2738) overexpression (NID1597). NID1597 showed decreased radial growth on MM + DOX, whereas NID1596 was not affected. Based on mRFP secretion in liquid cultures, it does not seem plausible that overexpression of either of the two proteins had a major effect on protein secretion. Overexpression of the *EMP47* ortholog AN7302 (NID1601) did not affect growth on plates or protein secretion in submerged cultivations.

In *S. cerevisiae,* results have shown that the expression levels of *ERV41* and *ERV46* are interdependent. Erv46p levels are lowered in an *erv41*Δ strain, and the Erv41p was not detected in an *erv46*Δ strain. Furthermore, the same study showed that overexpression of both proteins on 2μ plasmids did not result in higher expression of any of the proteins compared to a single overexpression of *ERV46.* Unaffected secretion in NID1596-1597 is in line with results from this study. Lastly, results have shown that expression of Erv41p was highly dependent on Erv46p, whereas Erv46p levels depended less on Erv41p [27]. We therefore speculate that it is possible that the growth effect seen on plates in strain NID1597 was due to the fact that overexpression of Erv46p resulted in concomitant increasing levels of Erv41p. Since the two proteins form a complex, this results in the observed growth effect due to higher levels of formed complexes. In NID1596, overexpression of *ERV41* might not result in increased Erv46p levels, thus resulting in a “normal” phenotype.

Recently, the *S. cerevisiae EMP47* ortholog *AoEmp47* was deleted and overexpressed in protein producing strains of *A. oryzae.* It was seen that deletion of *AoEmp47* improved heterologous protein production, whereas overexpression decreased secretion [28]. The reason for the decreased secretion upon overexpression of *AoEmp47* was that the protein is involved in retention of heterologous proteins in the ER. Our data with overexpression of *EMP47* ortholog in *A. nidulans* shows similar results as the control strain. Thus, there was no effect on secretion when overexpressing *EMP47* in *A. nidulans,* contrary to the results previously observed in *A. oryzae*.

### Overexpression of the rab GTPASE rabD significantly improves protein secretion

Overexpression of the rab GTPase *rabD* (NID1602) increased mRFP secretion by approximately 25 % in submerged cultivations in shake flasks (fluorescence units/g dw) (Additional file 2: Figure 2, DOX induced conditions). Hyphae of NID1602 appeared swollen compared to the reference strain, with an increased hyphal diameter and shorter compartment length observed (Fig. 5). The maximum dry weight in shake flask cultivations reached slightly lower levels when DOX was added to the media, however growth was not reduced to the same extent as on plates, where decreased radial growth was observed. Fluorescence microscopy revealed fluorescence was distributed towards the hyphal tips, plasma membrane and septa in NID1602 (Fig. 5).

**Fig. 6:**
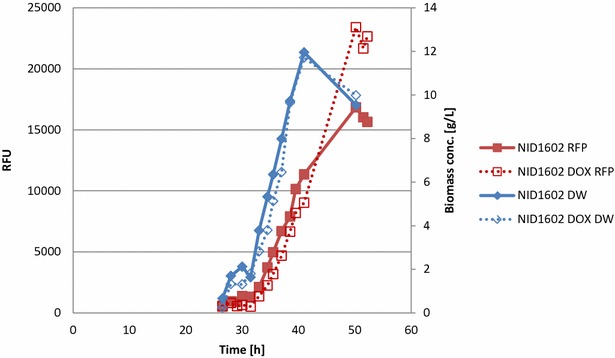
Growth and fluorescence of NID1602 in submerged cultivation in a 2 l bioreactor. Growth was measured as dry cell weight (g/L) and fluorescence in absorbance units. The data shown is for one representative culture of triplicates with a standard deviation in dry weight and fluorescence for all processes of less than 5 %

Submerged cultivation in 2L bioreactors was also performed with this strain, to provide controlled growth conditions with aeration and stirring profiles which were optimal for supporting fungal growth. In these experiments it was clearly shown that that the growth rate was not affected by the addition of DOX being 0.203 h^−1^ with and 0.212 h^−1^ with and without DOX addition, respectively (Fig. 6). Furthermore, RFP secretion in NID1602 was shown to be increased by 40 % (DOX induced compared to non-induced). This is a reflection of the more optimised and controlled growth conditions experienced in the bioreactor.

The *rabD* guanine nucleotide exchange factor AN4759 (*S.c.* S*EC2)* (NID1607) was chosen for overexpression to test whether it would have similar effects as the overexpression of *rabD,* since it functions as an activator of *rabD.* However, overexpressing AN4759 (NID1607) resulted in decreased protein secretion by approximately 30 %.

*RabD* is involved in vesicle transport from the Golgi to the plasma membrane, and the *A. niger* homolog *SrgA* has previously been found to influence protein secretion and morphology in *A. niger*. A deletion mutant showed decreased protein secretion as well as increased hyphal diameter during growth on glucose [7]. Unlike the situation in *S. cerevisiae*, it is not an essential gene for survival. In *A. fumigatus*, *srgA* deletion showed that the gene is involved in filamentous growth and asexual development. The deletion mutant also demonstrated increased susceptibility to Brefeldin A treatment, which inhibits vesicular trafficking in the cell [29]. In *A. fumigatus* SrgA localizes to the hyphal tip [29], and it can be speculated that the increased fluorescence in NID1602 hyphal tips was a result of more efficient transport of the secretory cargo towards the plasma membrane, which was also demonstrated in the fluorescence microscopy.

### Exocytosis

Overexpression of the t-SNARE protein SsoA resulted in unchanged secretion of the model protein in this study (NID1605). *S. cerevisiae* has two *SSO* genes, whereas *A. nidulans* has one. This suggests that there might be different roles of the proteins in the species. In *S. cerevisiae* overexpression of *SSO1* or *SSO2* has been shown to improve production of heterologous and homologous products [30].

### Overall influence on protein secretion by secretory pathway engineering

In order to look at qualitative effects on protein secretion in the engineered strains an SDS-PAGE was performed on selected strains with and without induction with DOX (Additional file 3: Figure 3). The strains were chosen to cover the range of effects seen previously on the fluorescence levels. The strains chosen were NID1439 (control), NID1600 (decreased fluorescence), NID1602 (increased fluorescence), NID1605 (unaltered fluorescence) and NID1609 (decreased fluorescence). Overall, adding DOX and the resulting overexpression of the gene candidates influenced the protein pattern in the supernatant. The control, NID1439, displayed a clear effect from adding DOX. However, looking carefully at the lanes with the supernatant from NID1600, NID1602, NID1605 and NID1609 without DOX addition, they resemble NID1439 with DOX addition in the type of bands present. Moreover, the sample in lane 2 appear to be less concentrated, as only the bands representing the heterologous proteins mRFP and the truncated GlaA (GlaA_1–514_) are visible, making it difficult to exclude an effect from adding DOX.

Samples from NID1600 and NID1609 (lanes 5 and 11), strains showing reduced fluorescence when their respective candidate genes were overexpressed, both displayed a lowering of secreted proteins except for the heterologous reporter proteins. In fact the ratio between the GlaA_1–514_ and mRFP seems to increase. The RabD overexpression strain (lanes 6 and 7) was the only strain that did not result in a relative decrease of endogenous proteins compared to the levels of mRFP and GlaA_1–514_. Also in this strain, the ratio between GlaA_1–514_ and mRFP seemed to increase in favour of GlaA_1–514_ levels. Interestingly, the strain displaying unaltered mRFP fluorescence levels without and with DOX addition (NID1605) also showed a decrease in endogenous protein levels in the overexpression strain (lane 9). This indicates that the overexpression strategy will give different outcomes for endogenous and heterologous proteins. The GlaA_1–514_ part of the fusion construct is generally seen in all overexpression strains after addition of DOX at 60 kDA (lanes 5, 7, 9, 11), whereas it hardly could be observed without addition of DOX (lanes 4, 6, 8, 10). This indicates that either GlaA_1–514_ secretion responds significantly and positively to all the gene overexpressions examined, or the effect is actually enhanced, or masked by a drop in secreted mRFP. Since we already saw efficient cleavage of the fusion protein, it suggests that the fates of the two heterologous proteins are different. For example, post-translational modifications, alternative transportation routes, retaining of glycoproteins (*e.g* GlaA) in the cell wall, and increased protease activity due to stress from the overexpression construct could be determinants that control the yields of the heterologous and endogenous proteins. Changes in the different parts of the secretory machinery appear to influence GlaA_1–514_ more than mRFP, and one significant difference is GlaA being glycosylated and, to our knowledge, mRFP not. Hence, glycosylated proteins could up to certain concentration be trapped in the cell wall, whereas less glycosylated protein would escape to the supernatant. Interestingly, the control strain cultivated without DOX acts differently from all other samples showing a relatively equal ratio between the two secreted proteins. It could point to either a biological meaning or just that small changes in cultivation conditions, especially in shake flask experiments, has variable impacts on different types of secreted proteins, and a controlled cultivation environment in bioreactors would be more suitable in future strategies.

Due to recent genome sequencing of filamentous fungal species, a cellular response to recombinant protein production is well documented and important proteins in this process well known. Nevertheless, reasons for the high secretion capacity of filamentous fungi are still relatively unknown, although some insights can be gained from studies on other microbial hosts, such as *S. cerevisiae*. As mentioned previously there has been a lot of attention towards accurate protein folding in fungal cells. Results are contradictory, and to some extent protein specific, indicating the complexity of the secretory pathway. In order to make use of available data and study the cellular response of manipulating the secretory pathway, this study has investigated processes that are involved in transport from or to different compartments. Results demonstrate that engineering the pathway leads to different secretion profiles for the fungal strains constructed, as well as differences in growth and morphology of the strains. As secretion modifications are likely to alter the transport of intracellular endogenous proteins it was not surprising that several of the modifications resulted in altered morphology [31]. The tet-ON promoter have previously been characterized by Meyer et al., and is an interesting tool for manipulation of genes that are important for the maintenance of cellular functions [20]. The promoter was therefore well suited for our study, as strain construction was facilitated by silencing the gene of interest to promote normal growth on transformation plates.

There are several reasons to why some of the genes overexpressed resulted in unchanged protein secretion in the constructed strains. In this study, one single copy of *glaA*_*1*–*514*_-fused mRFP was integrated, and this may not result in high enough throughput to saturate the system. It has previously been shown that increased gene copy number may result in increased secretion [32]. Thus, if the system is not saturated, overexpression of genes involved in translocation to the ER might not result in increased secretion of the model protein. In order to test if the system was saturated, we constructed a strain containing an additional copy of the *glaA*-*RFP* gene. This resulted in a 70 % increase in maximum fluorescence level (data not shown), demonstrating that there is indeed capacity for secreting higher amounts of the model protein, and this may lead to bigger impact when modifying the secretory pathway. Furthermore, protein dependent factors cannot be overlooked. For example, a more complex protein, where folding is more difficult and stressful to the cell, may lead to other bottlenecks within the secretory pathway than what can be observed with the mRFP protein alone.

An important factor for optimizing a protein cell factory is to relieve bottlenecks within the specific system that is being studied. The upregulation of r*abD* significantly boosted the secretion of the model protein, and it is possible that the bottlenecks for this strain now lie downstream of this gene, in the exocytosis step, or that overexpression of upstream genes will result in improved secretion due to the absence of the *rabD* bottleneck. Therefore, sequential overexpressions/deletions of a well-known system might be necessary in order to reach the full secretion potential of the host.

### Overexpression of genes

In order to verify that the addition of DOX induces overexpression the level of gene expression was measured with qPCR. The same strains evaluated with SDS-PAGE were chosen, namely NID1600 (decreased fluorescence), NID1602 (increased fluorescence), NID1605 (unaltered fluorescence) and NID1609 (decreased fluorescence). Gene expression was compared between samples with and without DOX. There was a clear increase in gene expression for all tested strains verifying that the genes were overexpressed in the strains when DOX was added, though the fold change varied from gene to gene (see Additional file 4: Figure 4).

## Conclusions

This study demonstrates the possibility of significantly increasing cellular recombinant protein secretion with approximately 40 % by overexpressing the Rab GTPase *rabD*. It is unlikely this is the only target for improving secretion, and further studies are likely to reveal additional candidates. Other gene targets investigated here, including genes from different compartments of the secretory pathway resulted in no significant change in protein secretion, or in significantly lowered protein titres. The overexpression of *AN6307 (S.c.* *SEC63* ortholog), the *A. niger An02g04250* ortholog AN6307 and the *rabD* GEF *AN4759* (*S.c SEC2* ortholog) resulted in substantially lowered titres of the recombinant protein. As the 14 genes selected in this study were previously shown to be upregulated during protein secretion, our results indicate that increased expression may be a way for the cell to slow down secretion in order to cope with the increased protein load, similarly to the observation for the gene *emp47* in other studies [28].

## Methods

### Strains

The *A. nidulans* strains used in this study are listed in Table 1. The *A. nidulans* strain IBT 29539 (*argB2, pyrG89, veA1, nkuA*Δ) (referred to as NID1) was used as parental strain for construction of mRFP secreting strain [33]. Plasmids were propagated in *E. coli* strain DH5α.

### Media and culture conditions

Minimal medium (MM), was used for cloning experiments and contained (per Liter): 50 mL nitrate salts solution, 1 mL trace element solution, 0.001 % thiamine, 10 g d-glucose.

Complex medium (CM) (per Liter), was used for bioreactor experiments and contained (per Liter): 2 g yeast extract, 3 g tryptone, 20 mL mineral mix solution, 10 g d-glucose, 0.1 M MES Buffer, pH 5.5.

20× nitrate salts solution (per Liter): 120 g NaNO_3_, 10.4 g KCl, 10.4 g MgSO_4_•7H2O, 30.4 g KH_2_PO_4._

50× mineral mix (per Liter): 26 g KCl, 26 g MgSO_4_•7H_2_O, 26 g KH_2_PO_4_, 50 mL trace element solution.

20× Trace element solution (per Liter): 0.4 g CuSO_4_•5H_2_O, 0.04 g Na_2_B_4_O_7_•10H_2_O, 0.8 g FeSO_4_•7H_2_O, 0.8 g MnSO_4_•2H_2_O, 0.8 g Na_2_MoO_4_•2H_2_O, 8 g ZnSO_4_•7H_2_O.

Plates and media were supplemented with doxycycline, l-arginine (0.7 g/L), Uracil (10 mM), Uridine (10 mM), sucrose (171,15 g/L) or 5-fluoroorotic acid (5-FOA, 1.3 mg/mL) as necessary during the molecular cloning procedures.

### Submerged cultivations

Shake flask cultivations were performed in 0.5 L Erlenmeyer flasks, without baffles, equipped with cotton stoppers. All cultivations were incubated at 30 °C with an agitation of 150 rpm. Spores were harvested in distilled water and filtered through a sterile miracloth and shake flasks were inoculated with 10^7^ spores/mL.

Batch cultivations were performed in 2 L volume glass Biostat B bioreactors (B. Braun Biotech) with a working volume of 1.6 L. All bioreactors were mounted with two six-bladed Rushton turbine impellers, pH electrode, thermosensor, sparger, sampling outlet and membrane port (for inoculation and addition of media supplements). CM was applied for all batch cultivations. Initial batch medium was sterilized in the bioreactors. Doxycycline was added through a sterile filter following sterilization, as required.

To minimize perturbation of cell growth, an automated procedure (ramp) adjusting process parameters was implemented. Initially, aeration was set to 0.1 vvm, agitation to 100 rpm, pH to 3 and temperature to 30 °C. Subsequently, assuming that spores were well germinated, aeration was increased to 2 vvm, agitation to 800 rpm and pH to 5. Temperature was kept constant throughout the cultivation. pH values were adjusted with 2 M NaOH and 2 M H2SO4. All bioreactor experiments were inoculated to a concentration of 10^9^ spores/mL (spore suspension prepared as above). Cultivations were carried out at least in duplicate.

### Molecular cloning

All PCR reactions were performed using the PfuX7 polymerase [34] in 35 reaction cycles with 60 °C annealing temperature and an extension time of 30 s/kb. All fragments relating to *A. nidulans* were amplified from *A. nidulans* NID1 gDNA. *A. niger* ATCC 1015 gDNA was used as template for amplification of glucoamylase (*glaA*) encoding gene. The plasmid pWJ1350 was used as template for amplification of *mRFP.* Primers, synthesized by Integrated DNA Technologies, are presented in Additional file 5: Table 1. Restriction enzymes and buffers were from New England Biolabs.

A list of all plasmids used in this study is presented in Additional file 6: Table 2. The plasmids for expressing *glaA*_1–514_ (aa 1–514 of *glaA*) fused mRFP [35] in *A. nidulans* from the *A. nidulans gpdA* promoter was constructed by fusing 6 individual DNA fragments with the vector backbone pU2002 [24]. The resulting plasmid was named pMAS1. To ensure proteolytic cleavage of the glucoamylase from the mRFP a KEX2 (Lys-Arg) proteolytic site was inserted between the glucoamylase and the mRFP protein. For purification, a C-terminal 6•His-tag was added to the mRFP. All plasmids were prepared for USER cloning by digesting with respective restriction and nicking enzymes, and the cloning procedure was as described in Nour-Eldin et al. [36].

To construct the plasmids for overexpression of secretion related genes, plasmid pU2311-1-ccdB was used. It contains the tetON promoter [20] which is induced by addition of doxycycline, *ampicillin* gene for selection in *E. coli,* *A. fumigatus pyrG (AFpyrG)* for selection in *A. nidulans* and up – and down-stream targeting sequences for integration in IS1 [24]. The secretion related genes were amplified from genomic DNA of *A. nidulans.* The constructed plasmids were named pMAS2-pMAS15.

### Genetic transformation

Protoplastation and transformation of *A. nidulans* were performed as described in Nielsen et al. [37] using AF*PyrG* as a selectable marker. Transformants were verified with PCR by using spores as the source of DNA. In order to liberate the DNA from the cells, the PCR mix with the spores was subjected to 20 min at 98 °C at the start of the PCR program. Then, a touchdown PCR program with annealing temperatures from 65 to 58 °C was performed. The spores were transferred to the PCR mix by gently touching a colony with the pipette tip and transferring the spores to two vials with the same reaction mix, ensuring that one of the reactions would have the correct amount of spores for DNA amplification.

The *A. nidulans* strain secreting mRFP was constructed by transforming NID1 with the linearized cassette from plasmid pMAS1 that integrates into a locus that has been previously used in our lab for high production of small metabolites. The cassette was liberated from the plasmid by treatment with SwaI restriction enzyme for 2 h at 25 °C. The transformation mix was plated on MM + Arg and transformants were verified by spore PCR. The constructed strain (NID1439) was streaked out on MM + Arg + Ura + Uri + 5-FOA in order to regenerate the marker by Direct Repeat recombination generating strain NID1595.

In order to construct secretion-related mutants the transformation cassette was liberated from pMAS2-15 by treatment with SwaI. The linearized cassette was transformed in to NID1595, and the transformants were verified for integration of the secretion related gene into integration site 1 (IS1, [24]). The constructed strains were named NID1596-NID1609. Furthermore, several strains were verified by southern blotting as described previously [38]. Four µg genomic DNA was digested with XhoI. The probe used for verification of integration of the gene in IS1 was generated by PCR. It was amplified with primers MS210 and MS211, and binds to the downstream fragment of IS1. The probe was labelled with Biotin-11-dUTP using the Biotin DecaLabelTM DNA Labeling kit (Fermentas). Detection was performed with the Biotin Chromogenic detection kit (Thermo scientific).

### RNA isolation and quantitative reverse transcription-PCR (qRT-PCR)

Samples from shake flask cultures in exponential growth were removed for determination of expression levels of the genes of interest, instantly frozen in liquid nitrogen and stored at −80 °C until analysis. The cells were disrupted using a Tissue-Lyser LT (Qiagen) by treating samples for 1 min at 45 MHz. Total RNA was isolated with the Qiagen RNeasy plus kit (Qiagen). The purity of the total RNA was determined spectrophotometrically using a NanoDrop Lite (Thermo Scientific). cDNA was made of total RNA using a QuantiTect Reverse Transcription Kit (Qiagen) according to the manufacturer’s instructions. The subsequent qRT-PCR was performed in a CFX Connect™ Real-Time PCR Detection System (Bio-Rad) by QuantiFast SYBR Green PCR Kit (Qiagen). PCR amplification was carried out in 20 µL reaction volume with the following cycle conditions: 95 °C for 5 min and 40 cycles of 95 °C for 10 s and 60 °C for 30 s. A melting curve from 65 °C to 95 °C with reads every 0.15 min was generated at the end of the program to evaluate the specificity of the PCR products. The *A. nidulans* gamma actin gene *actA* (AN6542) was used as the internal standard for normalization of expression levels. All primers used for qRT-PCR are shown in Additional file 5: Table 1. The relative expression levels were approximated based on 2ΔΔCq, with ΔΔCq = ΔCq(normalized)−ΔCq(calibrator), where ΔCq(normalized) = ΔCq(target gene)−ΔCq(actA). The calibrator Cq values are those from the strains without DOX.

### Cell dry weight determination

Cell dry weight was determined by filtering of cell culture through a pre-dried and weighed filter (Advantec). The filter was dried and weighed again, and the dry weight was determined by calculating the amount of dry cell weight per liter of cell culture.

### Fluorescence measurement

Fluorescence of culture filtrates were measured in a Synergy Mx Monohromator-Based Multi-Mode Microplate Reader (BioTek Instruments) using excitation/emission 584/607 nm. A 96-well microtiter plate (PS microplate, Greiner bio-one) was used and 200 µL samples were loaded in triplicates. Background fluorescence was corrected by subtraction of values derived from a negative control.

### SDS-PAGE

SDS-PAGE was performed on Novex NuPAGE 4–12 % Bis-Tris gel (Life Technologies) according to the instructions of the manufacturer. The ladder used was Novex Sharp Pre-stained Protein Standard (Life Technologies).

### Upconcentration of supernatant

Culture supernatant was upconcentrated using Amicon^®^ Ultra 0.5 mL centrifugal filter unit with ultracel-10 membrane (Merck Millipore). Purification of His-tagged mRFP was performed with a His SpinTrap kit (GE Healthcare).

### Microscopy

MM agar slides were prepared by pipetting 1 ml agar containing MM. MM agar slides were inoculated with spores and grown at 30 °C in petri dishes until analysis. Live cell images were captured with a cooled Evolution QEi monochrome digital camera (Media Cybernetics Inc.) mounted on a Nikon Eclipse E1000 microscope (Nikon). Images were captured using a Plan-Fluor ×100, 1.30 numerical aperture objective lens. The illumination source was a 103-watt mercury arc lamp (Osram). The fluorophore mRFP was visualised using a band pass RFP filter (EX545/30, EM620/60 combination filter; Nikon). Each slide was scanned manually, and representative images were captured to document the morphological phenotype and fluorescence pattern of each strain. Red colour was added to each image where a fluorescence signal was obtained using image processing in ImageJ.

## Additional files

**Additional file 1: Figure 1.** Verification of integration of secretory genes in IS1 by southern blot. A and B: Arrows indicate XhoI cut sites, and the resulting sizes of the fragments are shown. C: Southern blot of strains NID1439, NID1599, NID1600, NID1602, NID1604, NID1605, NID1608 and NID1609 digested with XhoI and hybridized with probe binding to downstream region of IS1. The illustration is not drawn to scale.

**Additional file 2: Figure 2.** Dry weight measurements (above) and fluorescence per g dw (below) over time in NID 1602 cultivated in shake flasks at 150 rpm at 30 °C.

**Additional file 3: Figure 3.** SDS-PAGE of supernatant from shake flask cultures. The supernatant was up-concentrated approximately 40x. 10 μL of the up-concentrated supernatant was loaded to each well. 1: Ladder, 2: NID1439, 3: NID1439DOX, 4: NID1600, 5: NID1600DOX, 6: NID1602, 7: NID1602DOX, 8: NID1605, 9: NID1605DOX, 10: NID1609, 11: NID1609 DOX, 12: Ladder.

**Additional file 4: Figure 4.** Relative fold change in gene expression level for 4 key genes (with DOX relative to without DOX).

**Additional file 5: Table 1.** Primers used in this study.

**Additional file 6: Table 2.** Plasmids used in this study.
