# Supplementary figures and images for: Effect of secretory pathway gene overexpression on secretion of a fluorescent reporter protein in Aspergillus nidulans
Source: Fungal Biol Biotechnol. 2016 Apr 12;3:3. doi: 10.1186/s40694-016-0021-y (PMC5611598; doi:10.1186/s40694-016-0021-y)

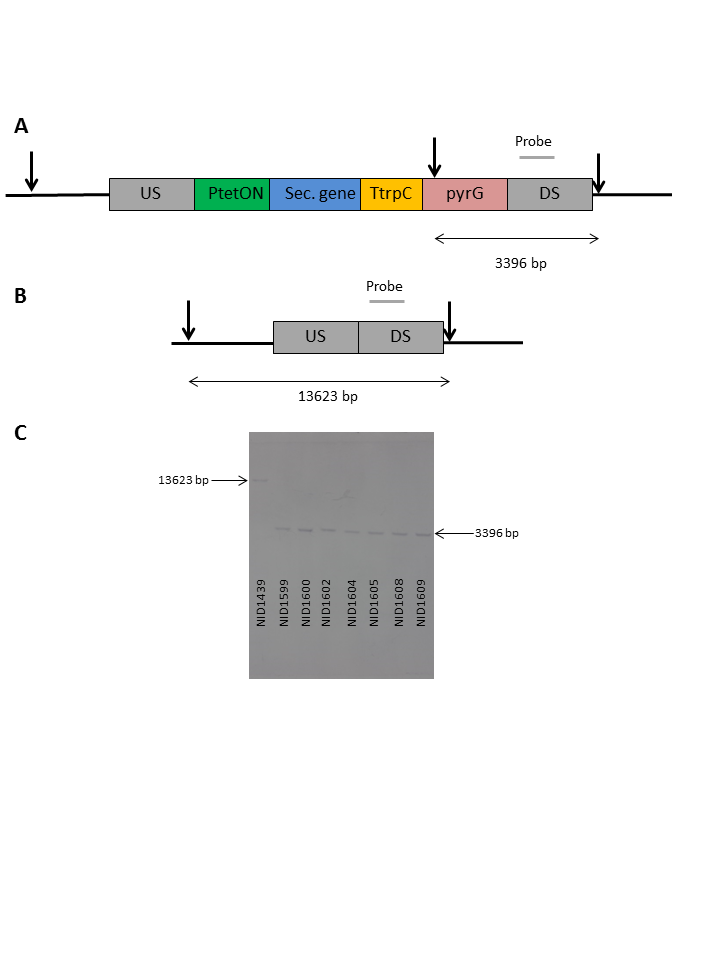
Supplementary Figure 1

Supplement: Supplementary file 1 — Additional file 1: Figure 1. Verification of integration of secretory genes in IS1 by southern blot. A and B: Arrows indicate XhoI cut sites, and the resulting sizes of the fragments are shown. C: Southern blot of strains NID1439, NID1599, NID1600, NID1602, NID1604, NID1605, NID1608 and NID1609 digested with XhoI and hybridized with probe binding to downstream region of IS1. The illustration is not drawn to scale. [file 40694_2016_21_MOESM1_ESM.docx]

## Slide 1
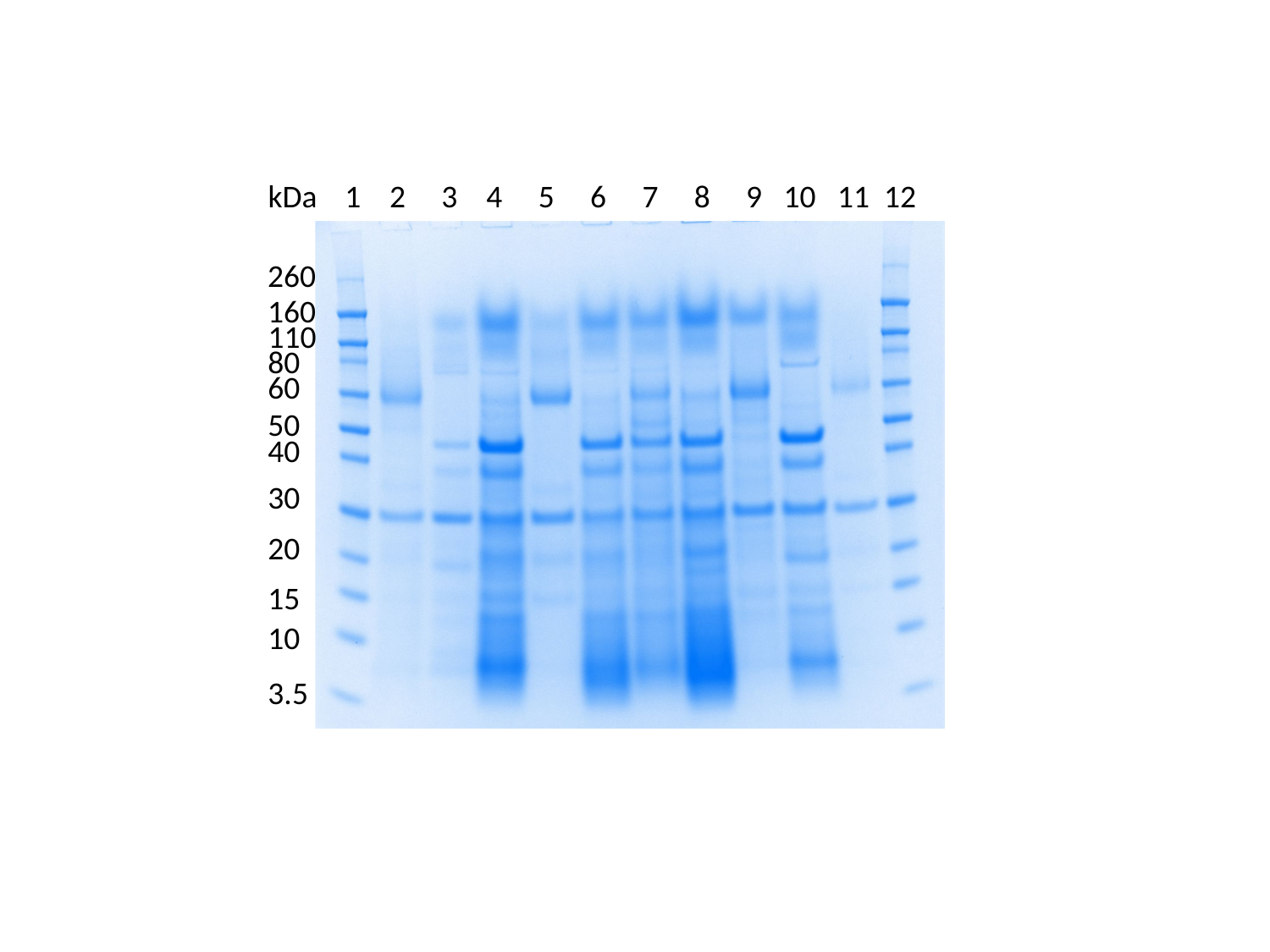

1 2 3 4 5 6 7 8 9 10 11 12
kDa
260
160
110
80
60
50
40
30
20
15
10
3.5

Supplement: Supplementary file 3 — Additional file 3: Figure 3. SDS-PAGE of supernatant from shake flask cultures. The supernatant was up-concentrated approximately 40x. 10 μL of the up-concentrated supernatant was loaded to each well. 1: Ladder, 2: NID1439, 3: NID1439DOX, 4: NID1600, 5: NID1600DOX, 6: NID1602, 7: NID1602DOX, 8: NID1605, 9: NID1605DOX, 10: NID1609, 11: NID1609 DOX, 12: Ladder. [file 40694_2016_21_MOESM3_ESM.pptx]
