# Supplementary material for: Effect of secretory pathway gene overexpression on secretion of a fluorescent reporter protein in Aspergillus nidulans
Source: Fungal Biol Biotechnol. 2016 Apr 12;3:3. doi: 10.1186/s40694-016-0021-y (PMC5611598; doi:10.1186/s40694-016-0021-y)
Supplement: Supplementary file 2 [file 40694_2016_21_MOESM2_ESM.docx]

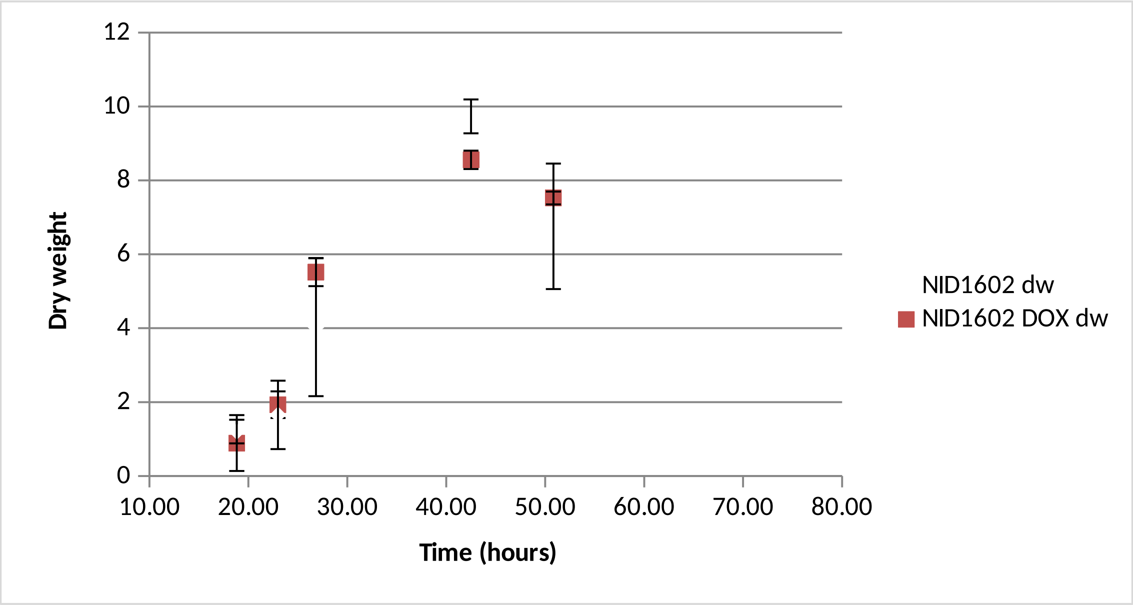
Dry weight
12
10
8
6
4
2
0
10.00
20.00
30.00
40.00
50.00
60.00
70.00
80.00
Time (hours)
NID1602 dw
NID1602 DOX dw

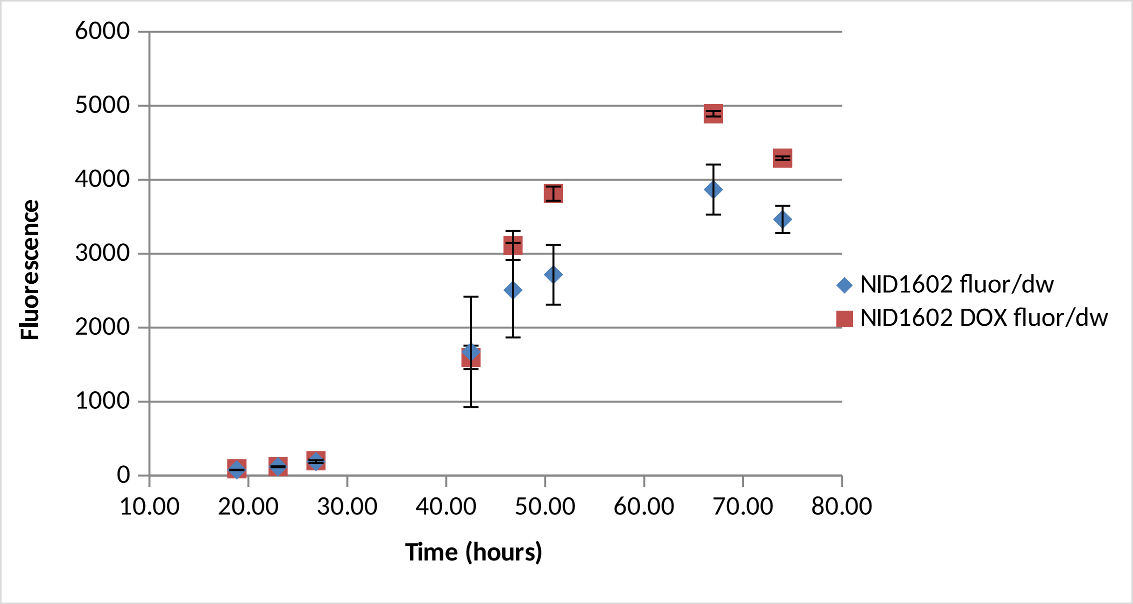
Fluorescence
6000
5000
4000
3000
2000
1000
0
10.00
20.00
30.00
40.00
50.00
60.00
70.00
80.00
Time (hours)
NID1602 fluor/dw
NID1602 DOX fluor/dw
