# Supplementary material for: Effect of secretory pathway gene overexpression on secretion of a fluorescent reporter protein in Aspergillus nidulans
Source: Fungal Biol Biotechnol. 2016 Apr 12;3:3. doi: 10.1186/s40694-016-0021-y (PMC5611598; doi:10.1186/s40694-016-0021-y)
Supplement: Supplementary file 3 [file 40694_2016_21_MOESM4_ESM.docx]

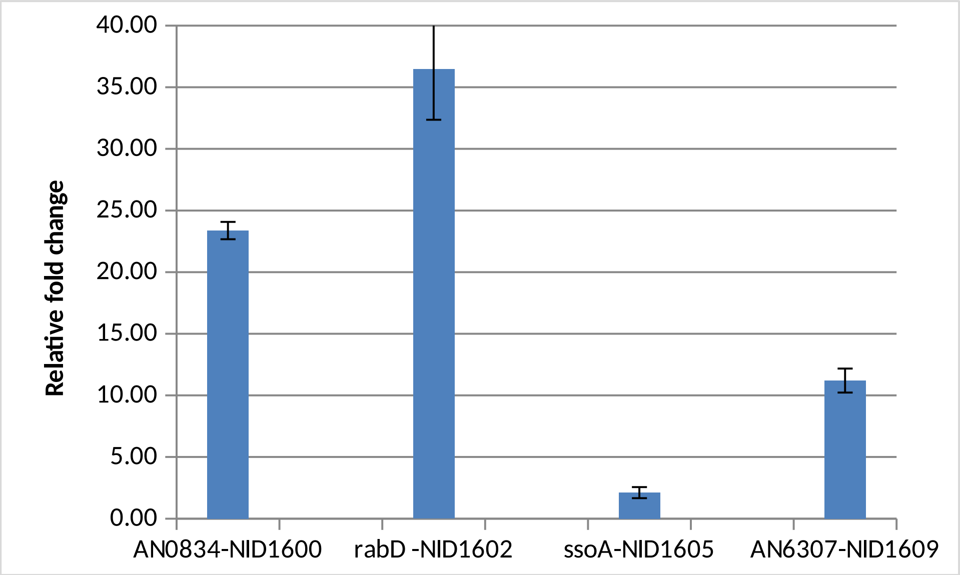

40.00
35.00
30.00
25.00
20.00
15.00
10.00
5.00
0.00
Relative fold change
AN0834-NID1600
rabD -NID1602
ssoA-NID1605
AN6307-NID1609
